# Rb is required for retinal angiogenesis and lamination

**DOI:** 10.1038/s41419-018-0411-6

**Published:** 2018-03-06

**Authors:** Yi Zhou, Ran Wei, Liu Zhang, Yongjiang Chen, Suying Lu, Chen Liang, Yujiao Wang, Lirong Xiao, Junjun Zhang, Rod Bremner, Danian Chen

**Affiliations:** 1 Research Laboratory of Ophthalmology and Vision Sciences, Torsten-Wiesel Research Institute of World Eye Organization, State Key Laboratory of Biotherapy, West China Hospital, Sichuan University, Chengdu, China; 2 Department of Ophthalmology, West China Hospital, Sichuan University, Chengdu, China; 3 Lunenfeld-Tanenbaum Research Institute, Sinai Health System, Toronto, ON Canada; 4 Departments of Ophthalmology and Visual Science, and Laboratory Medicine and Pathobiology, University of Toronto, Toronto, ON Canada

## Abstract

Retinoblastoma tumor suppressor (Rb) promotes cell cycle exit, survival, differentiation, and tumor suppression in the retina. Here, we show it is also essential for vascularization and lamination. Despite minimal effects on Hif1a target expression, intraretinal vascular plexi did not form in the *Rb*^*−/−*^ murine retina. Deleting adenovirus E2 promoter binding factor 3 (E2f3), which rescues starburst amacrine cell differentiation, or E2f2, had no effect, but deleting E2f1, which promotes neuronal cell cycle exit and survival, restored retinal vasculature. We specifically linked cell loss to the defect because removing Bax rescued rod and bipolar neurons and the vasculature, but not cell cycle exit. Despite rescuing *Rb*^*−/−*^ neurons, *Bax* deletion exacerbated a delay in outer retina lamination, and exposed a requirement for Rb in inner retina lamination. The latter resembled *Sem5* or FAT atypical cadherin 3 (*Fat3*) mutants, but expression of Sem5/Fat3 pathway components, or that of Neogenin, which perturbs migration in the *Rb*^*−/−*^ cortex, was unchanged. Instead, lamination defects correlated with ectopic division, and were E2f1-dependent, implicating the cell cycle machinery. These in vivo studies expose new developmental roles for Rb, pinpoint aberrant E2f1 and Bax activity in neuronal death and vascular loss, and further implicate E2f1 in defective lamination. Links between Rb, angiogenesis and lamination have implications for the treatment of neovascularization, neurodegeneration and cancer.

## Introduction

Angiogenesis is a critical step in development and disease and is regulated by pro-angiogenic and anti-angiogenic factors^1^. In mice, the retinal vasculature consists of three interconnected parallel vascular plexi. A superficial vascular plexus (SVP) in the nerve fiber layer (NFL) develops from the optic nerve head and progresses radially to the peripheral retina between postnatal day 0 (P0) and P8. Subsequently, vessels sprout vertically into the retina. Around P7, sprouting vessels descend and advance into the outer plexiform layer (OPL) to establish the deep vascular plexus (DVP). Around P11, the DVP vessels ascend into the inner plexiform layer (IPL) and form the intermediate vascular plexus (IVP)^2–4^. Previous studies revealed that Frizzled-4 (Fzd4), Lrp5, Norrin, and Tetraspanin 12 (Tspan12) are required for intraretinal vascular development^3,5,6^, and retinal Hif1a is required for IVP development^7^. Retinal neurons form the neurovascular unit to interact with endothelial cells^4,8^. Retinal ganglion cells (RGCs) are essential for the SVP development^9,10^, photoreceptors are important for the development of the intraretinal vascular plexus^11,12^, and amacrine and horizontal cells are critical for developing and maintaining the intraretinal vasculature^4^.

The retinoblastoma tumor suppressor (Rb) plays a major role in regulating cell cycle and other cellular processes by interacting with adenovirus E2 promoter binding factors (E2fs)^13^. The Rb/E2f pathway plays critical roles in angiogenesis^1^. For example, Rb binds Hif-1α and enhances its transcriptional activity^14^. Atypical E2fs (E2F7/8) bind Hif-1α to stimulate Vegfa induction^15^. As such, deletion of *Rb1*, or *E2f7/8*, or *Hif1* in mice all result in vascular defects in the placenta and early embryonic lethality^16–18^.

The retina comprises three distinct nuclear layers (GCL, ganglion cell layer; INL, inner nuclear layer; and ONL, outer nuclear layer), separated by two synaptic layers (OPL and IPL). Retinal lamination is guided by many different cues^19^, such as Dscam^20,21^, FAT atypical cadherin 3 (Fat3)^22^, semaphorins (Sema), and plexins^23^. Proper development of retinal lamination is important for physiological retinal responses and function. Recent studies have revealed that Rb can regulate neuronal migration and cortical lamination^24,25^.

Previously we reported that Pax6 alpha enhancer Cre (*α-Cre*)-mediated *Rb* gene knockout (KO) in mouse retina cause ectopic cell division, cell death, and differentiation defects^26,27^. *α-Cre* is active from embryonic day 10 (E10) in the peripheral progenitors of the temporal and nasal retina^28,29^. Employing this model we now implicate Rb in formation of the two intraretinal vascular plexi, as well as lamination of the outer and inner retina. We show that the angiogenesis defect is related to E2f1 and Bax-induced retinal cell death, whereas the lamination defects are cell death-independent and instead correlate with ectopic cell division.

## Results

### *Rb* is required for the development of intraretinal capillaries and retinal lamination

**Fig. 1:**
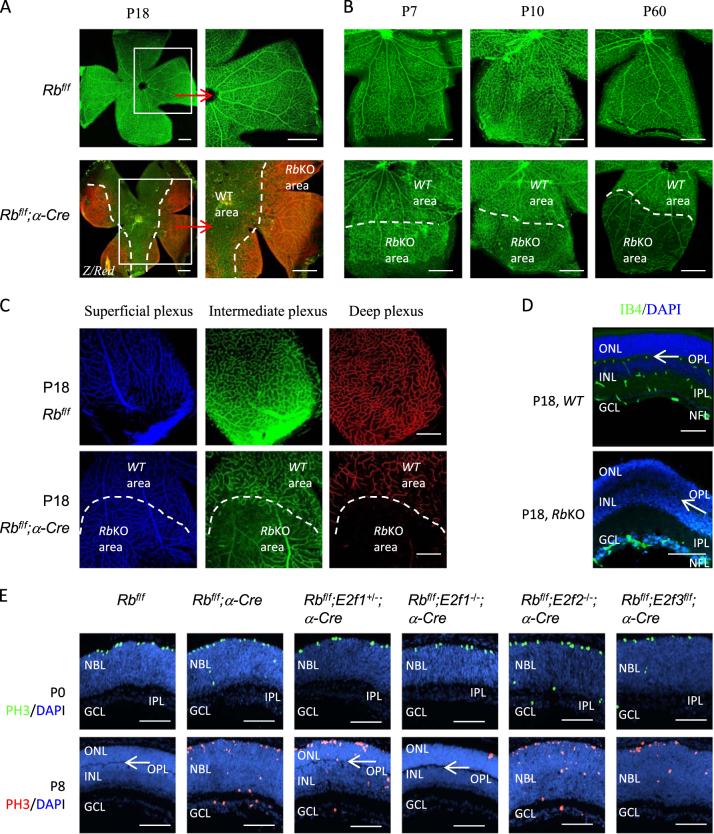
*Rb* is required for the development of intraretinal vascular plexi and the OPL. **a** Isolectin B4 (IB4) staining of P18 whole-mount retinas of *Rb*^*f/f*^ (*WT* control) and *Rb*^*f/f*^;*Z/Red;a-Cre* (*RbKO* in red areas) mouse. Selected areas are blown up to show the vascular density. **b** Whole-mount retinas of the indicated ages and genotypes were stained for IB4. **c** Confocal images of IB4-stained SVP, IVP, and DVP of P18 whole-mount retinas of the indicated genotypes. Pseudo-colors were used to differentiate these three plexi. **d** IB4 (green) and DAPI (blue) staining of P18 retinal sections of *WT* or *Rb*KO retina. **e** Horizontal sections of indicated ages and genotypes were stained for nuclear (DAPI, blue), mitosis (PH3, green at P0, red at P8). Arrows in **d** and **e** indicate the position of outer plexiform layer (OPL). The dotted lines in **a**–**c** indicate the boundary between *WT* (in the center) and *Rb*KO areas (in the periphery). NBL neuroblast layer, ONL outer nuclear layer, INL inner nuclear layer, IPL inner plexiform layer, GCL ganglion cell layer. Scale bar is 50 µm

We examined *Rb*^*f/f*^;*α-Cre* and *Rb*^*f/f*^ retinas between P7 and P60. To mark the *Rb*KO area, we utilized Cre reporter *Z/Red* mice which express the red fluorescent protein upon Cre-mediated recombination^30^. Retinal whole-mount staining revealed that at P18 and later time points, the density of retinal blood vessels was much lower in the peripheral *Rb*KO area of *Rb*^*f/f*^;*α-Cre* retinas than that in the same areas of the *Rb*^*f/f*^ retinas (wild-type (*WT*) control), and the *WT* areas of *Rb*^*f/f*^;*α-Cre* retinas (Fig. 1a). Killing RGCs in the embryonic retina blocks the SVP formation in the postnatal tissue, as does Math5 deletion, which prevents the genesis of 95% RGCs^9,10^. Surprisingly, in the *Rb*^*f/f*^;*α-Cre* retina, where 80% RGCs are deleted in the P0 peripheral retina^26^, the SVP formed normally (Fig. 1b–d). This finding suggests that the reduced RGC density in the periphery and/or that the RGC signals in the central retina are sufficient to promote peripheral vascular outgrowth. The SVP remained intact at later stages (Fig. 1b–d), thus peripheral RGCs are also not required to maintain these vessels. At P18 the *Rb*KO area had a normal SVP, but almost entirely lacked the IVP and DVP (Fig. 1c), as confirmed by histological staining of retinal sections (Fig. 1d). In *WT* P18 retinal sections, isolectin B4-positive (IB4^+^) cells were found in the NFL, IPL, and OPL, corresponding to the SVP, IVP, and DVP, respectively. However in *Rb*KO retinal sections, IB4^+^ cells were only found in the NFL, but not in the IPL and OPL (Fig. 1d). In the P60 *Rb*KO retina, the IVP and DVP were still absent, indicating that the defect was not transient due to delayed development (Fig. 1b).

We also examined the lamination of *Rb*-deficient retinas. At P0, the IPL had already formed between neuroblast layer (NBL) and GCL in *WT* and *Rb*^*−/−*^ retinas (Fig. 1e); thus, although *Rb* is required for maturation of a subset of amacrine cells^27^, most of the IPL forms in its absence. At P8, the OPL had formed between the ONL and INL, in the *WT* but not *Rb*^*−/−*^ retina (Fig. 1e). At P18, the OPL had also formed in the *Rb*^*−/−*^ retinas (Fig. 1d), suggesting that *Rb* deficiency delayed OPL formation, likely due to fewer rod and bipolar cells^26^. This phenotype is similar to the migration and lamination defects in the developing *Rb*^*−/−*^ cortex^25^. We concluded that *Rb* is required for the development of intraretinal capillary plexi and formation of the OPL.

### A subset of angiogenesis regulators is down-regulated following *Rb* loss

The α-Cre transgene is restricted to retinal cells, indicating a non-cell autonomous effect of Rb loss on angiogenesis. As the DVP and IVP originate from the SVP at about P7 and P11^2,4,31^, respectively, the defects should begin at around P7. Notably, P7 is the peak time for ectopic cell division and cell death of the *Rb*^*−/−*^ retina^26^. As Rb can bind Hif-1α and enhance its transcriptional activity^14^, *α-Cre*-mediated *Hif-1α* KO retina lacks the IVP^7^, *Rb* loss may directly reduce Hif-1α activity, and thus the vascular defects. However, gene expression analysis did not support this notion.

**Fig. 2:**
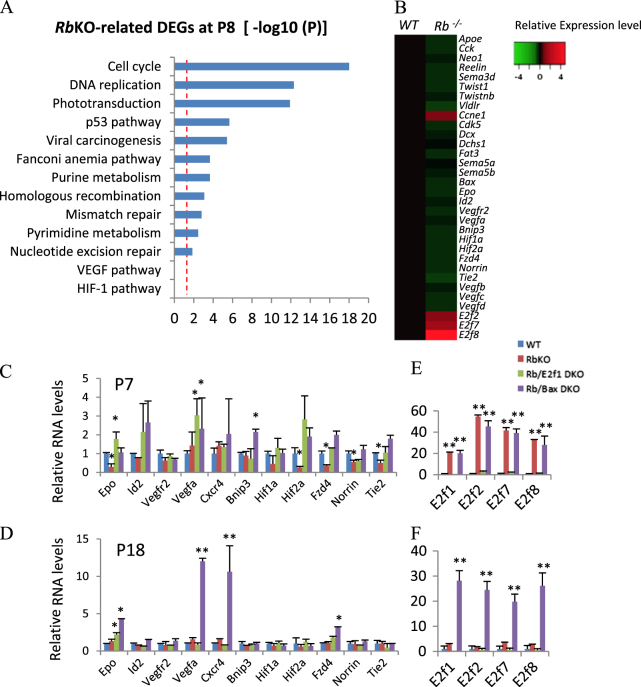
Gene expression changes in the *Rb*-null retina and the effect of E2f1 or Bax loss. **a** Gene list enrichment analysis using Kyoto Encyclopedia of Genes and Genomes (KEGG) 2016 datasets in Enrichr of *Rb*KO-regulated retinal DEGs at P8 (−log 10(*P*)). Dotted line indicates adjusted *p* < 0.05. **b** Heatmap of relative expression level of selected genes of P8 *Rb*KO and *WT* retina, based on the microarray analysis. **c**–**f** Real-time RT-PCR analysis of angiogenesis genes (**c**, **d**) and the E2f family (**e**,** f**) at P7, P18 retinas of the indicated genotypes, respectively. Error bars represent SD of measurements from three animals, and asterisks indicate a significant difference between the *WT* and the indicated genotypes (*p < 0.05; **p < 0.01, one-way ANOVA followed by Bonferroni correction)

We identified 677 *Rb*KO-related deregulated genes (DEGs) from the microarray data of P8 *Rb*KO retinas (Gene Expression Omnibus (GEO) accession: GSE86372)^32^. Gene enrichment analysis by Enrichr^33,34^ indicated that the most enriched pathways of *Rb*KO-related DEGs included the cell cycle and DNA replication, consistent with Rb function, and phototransduction, explained by rod death, but not the Hif1 or Vegf pathways (Fig. 2a, b). Reverse transcription-polymerase chain reaction (RT-PCR) revealed that, compared to *WT* retinas, while *Epo* was reduced in *Rb*^*−/−*^ retina at P7, other Hif-1α targets such as *Vegfa*, *Id2*, *Vegfr2*, *Bnip3*, and *Cxcr4* were unchanged at P7 and P18 (Fig. 2c, d). One possibility is that the *Rb*^*−/−*^ retina has higher expression of *E2f7* and *E2f8* (Fig. 2b, e, f)^27^, which can enhance Hif-1α transcriptional activity^35^, thus compensating for the effect of *Rb* loss on *Hif1a* activity. On the other hand, non-Hif1a targets such as *Norrin*, *Fzd4*, and *Tie2*, which are important for intraretinal vascular capillaries^2,6^, were reduced in *Rb*^*−/−*^ retina at P7 (Fig. 2b, c).

### Inactivating E2f1, but not E2f2 or E2f3, rescues the vascular and lamination defects of *Rb*KO retina

**Fig. 3:**
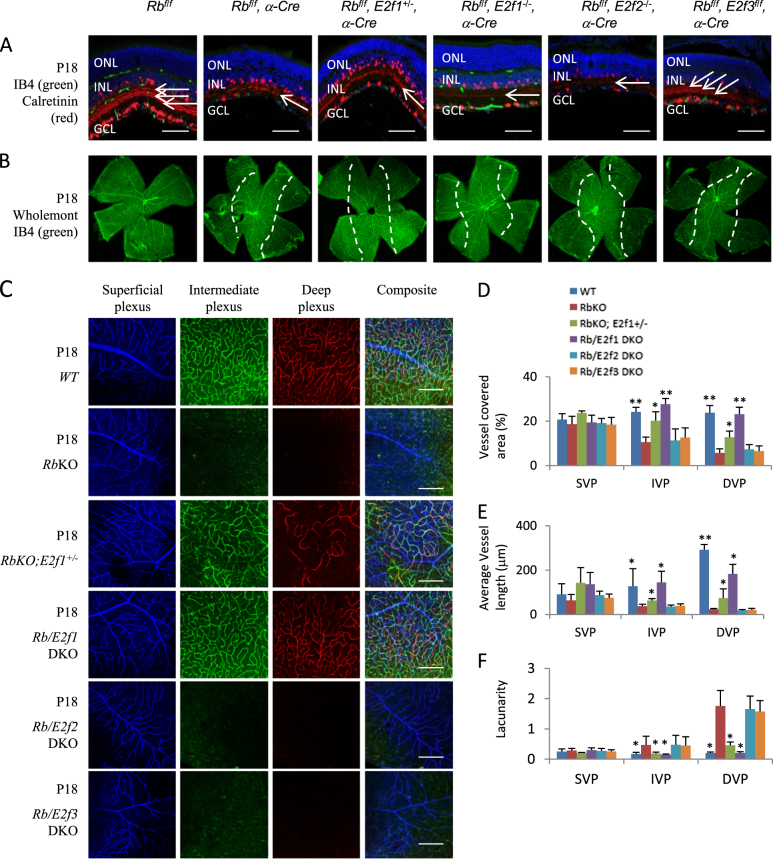
E2f1 mediates the *Rb*KO-induced retinal angiogenesis and lamination defects. **a** P18 retinal sections of the indicated genotypes were stained for nuclear (DAPI, blue), vascular endothelium cells (IB4, green), and amacrine cells (Calretinin, red). White arrows indicate Calretinin^+^ tracks in the IPL; loss of the outer two tracks reveals the starburst amacrine cell defect. **b** P18 whole-mount retinas of indicated genotypes were stained for IB4 to label vasculature. Dotted lines indicate the boundary between *WT* (center) and *Rb*KO (peripheral) areas. **c** Confocal images of IB4-stained SVP, IVP, and DVP of P18 whole-mount retinas of indicated genotypes. Pseudo-colors were used to differentiate these three plexi. **d**–**f** Quantification of vessel coverage (**d**), average vessel length (**e**), and lacunarity (**f**) by the AngioTool software. Error bars represent SD of measurements from at least three animals and asterisks indicate significant differences between retinas of *Rb*KO and the indicated genotypes (*p < 0.05, **p < 0.01, one-way ANOVA followed by Bonferroni correction). Scale bar is 50 μm. ONL outer nuclear layer, INL inner nuclear layer, GCL ganglion cell layer, ON optic head

E2f1 can suppress^36^ or promote^37^ angiogenesis in different conditions; however, we have not found any retinal vascular or lamination defects in *E2f1KO*, or *E2f2KO*, or *E2f3*^*f/f*^;*α-Cre* mice. We reported that in the *Rb*KO retina, E2f1 mediates ectopic division and cell death, E2f3 disrupts starburst amacrine cell (SAC) differentiation, and while E2f2 does not cause defects in the *RbKO* retina, it mediates the ectopic division and death of cones of *Rb/p107* double KO (DKO) retina^27,38,39^. To define whether any *E2f* contributes to the vascular defects, we crossed *Rb*^*f/f*^;*α-Cre* mice with *E2f1*^*−/−*^, or *E2f2*^*−/−*^, or *E2f3*^*f/f*^ mice (Fig. 3a, b).

We used the AngioTool software to analyze the vessel coverage, average vessel length, and lacunarity of vascular plexi. This analysis confirmed that the *Rb*KO retina had a normal SVP, but the vascular density and average vessel length were much reduced in IVP and DVP (Fig. 3c–f). Deleting *E2f3* rescued Calretinin^+^ SAC processes in the *Rb*^*−/−*^ IPL (Fig. 3a), as before^27^, but neither that nor E2f2 loss affected angiogenesis (Fig. 3b–f). In stark contrast, removing *E2f1*, which does not rescue the SAC defect (Fig. 3a), completely reversed the loss of both IVP and DVP, and returned vessel coverage and length as well as lacunarity to *WT* levels (Fig. 3b–f). Even removing one *E2f1* allele had a marked effect (Fig. 3b–f). *E2f1*KO also restored *Fzd4* and *Tie2* mRNA levels in the *Rb*^*−/−*^ retina at P7 (Fig. 2b), which may account for the rescue of retinal vascular defects. These data are reminiscent of the dose-dependent effects of *E2f1* on abnormal cell division and death in the *Rb*KO retina^38^.

The lamination defects were rescued by either *E2f1*^*+/−*^ or *E2f1*^*−/−*^, but neither *E2f2* nor *E2f3* loss. These results indicate that whereas E2f2 or E2f3 activity, or disrupted SAC differentiation are not involved, E2f1 drives the vascular and lamination defects in the *Rb*KO retina. This is slightly different from the developing *Rb*^*−/−*^ cortex, in which either *E2f1*^*−/−*^ or *E2f3*^*−/−*^ rescues the lamination defects^25,40^. E2f1 drives both ectopic division and cell death in the *Rb*KO retina^27^, thus we next sought to define which of these cellular defects contributes to vascular disruption and retinal lamination defects.

### Bax contributes to cell death and retinal vasculature defects

**Fig. 4:**
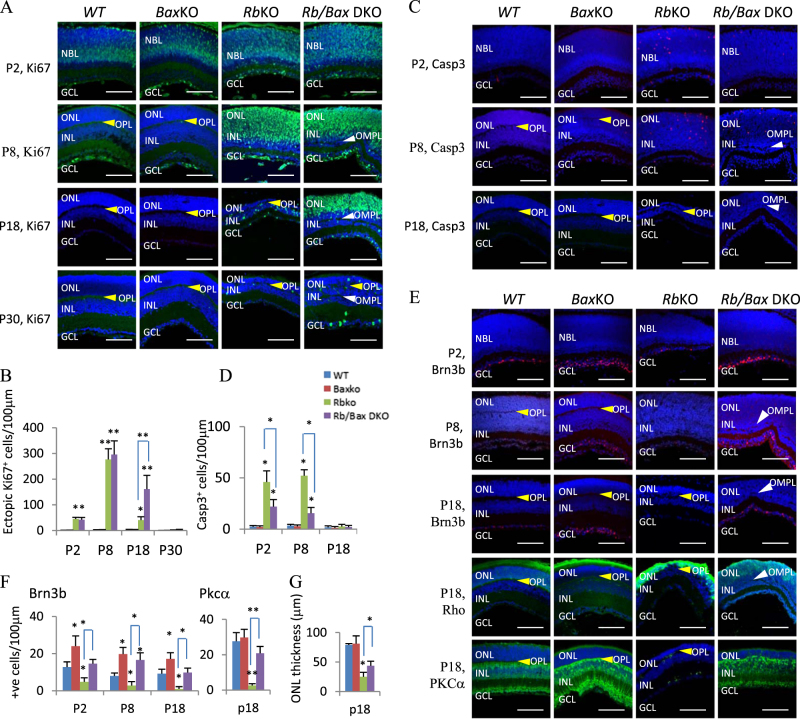
Bax loss rescues *Rb*KO neurons. **a** Retinal sections of indicated ages and genotypes were stained for nuclei (DAPI, blue) and division (Ki67, green). **b** Quantification of data in **a**. **c** Sections were stained for nuclei (DAPI, blue) and apoptosis (Casp3, red). **d** Quantification of data in **c**. **e** Sections were stained for nuclei (DAPI, blue), ganglion cells (Brn3, red), rod bipolar cells (PKCα, green), or rod photoreceptors (Rho, green). **f**, **g** Quantification of the data in **e**. White and yellow arrowheads in **a**, **c**, **e** highlight the OPL and OMPL, respectively. Data in **b**, **d**, **f**, **g** are mean ± SD. Asterisks indicate significant difference between *WT* and other genotypes, or between *Rb*KO and *Rb/Bax* DKO as indicated by square brackets (*p < 0.05, **p < 0.01, one-way ANOVA followed by Bonferroni correction). ONL outer nuclear layer, INL inner nuclear layer, GCL ganglion cell layer, OPL outer plexiform layer, OMPL outer misplaced plexiform layer. Scale bar is 50 µm

The Bcl-2 family member Bax mediates neuronal apoptosis^41^, including physiological retinal apoptosis^42,43^ and neuronal death in the *Rb/p107* DKO brain^24^. Thus, we tested whether Bax drives cell death in the *Rb*KO retina. *Bax*^*−/−*^ did not affect ectopic division in the *Rb*KO retina at P2 or P8, and actually elevated proliferation at P18 (Fig. 4a, b). All ectopic division had ceased by P30 indicating Rb-independent means of cell cycle exit. The increase in dividing cells at P18 might result from improved survival of ectopically proliferating *Rb*^*−/−*^ cells at earlier times, and indeed apoptosis was markedly reduced in the *Rb/Bax* DKO retina at P2, and P8 when cell death peaks (Fig. 4c–d). Thus, Bax drives cell death in the *Rb*^*−/−*^ retina.

Next, we defined which death-prone *Rb*^*−/−*^ cell types are rescued by *Bax*KO. Rb loss causes death of most rod bipolar and RGCs, and many rods^26^. Bax loss restored RGCs to *WT* numbers, and also suppressed rod bipolar cell death considerably (Fig. 4e, f). Rhodopsin staining and measurement of ONL thickness indicated partial rescue of rod photoreceptors (Fig. 4e, g). Thus, Bax is a major, but not the sole contributor to apoptosis in *Rb*^*−/−*^ neurons. Its role is greater in the inner (ganglion, bipolar cells) than outer retina (photoreceptors).

**Fig. 5:**
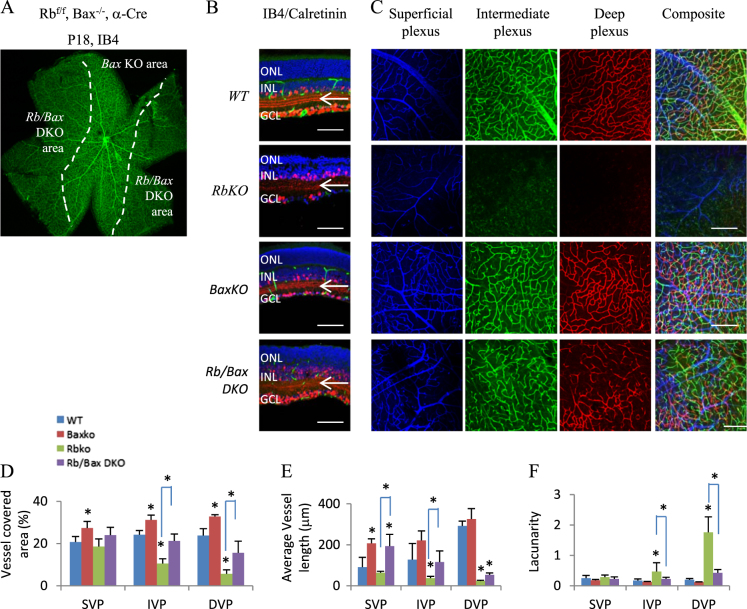
*Bax*KO ameliorates the vascular defect in the *Rb*KO retina. **a** Isolectin B4 (IB4) staining of P18 whole-mount retinas of *Rb*^*f/f*^;*Bax*^*−/−*^*;a-Cre* mouse. **b** P18 retinal sections of indicated genotypes were stained for nuclei (DAPI, blue), vascular endothelial cells (IB4, green), and amacrine cells (Calretinin, red). Arrows highlight the amacrine neurite tracks in the IPL. ONL outer nuclear layer, INL inner nuclear layer, GCL ganglion cell layer. **c** Confocal images of IB4-stained superficial, intermediate, and deep plexi of P18 whole-mount retinas of indicated genotypes. Pseudo-colors were used to differentiate the three plexi. **d**–**f** Quantification of vessel coverage (**d**), average vessel length (**e**), and lacunarity (**f**) by the AngioTool software. Error bars represent SD of measurements from at least three animals and asterisks indicate significant difference between *WT* and other genotypes, or between *Rb*KO and *Rb/Bax* DKO as indicated by square brackets (*p < 0.05, one-way ANOVA followed by Bonferroni correction). Scale bar in **b** and **c** is 50 µm

Next, we asked whether *Bax*KO affects the disrupted vasculature of the *Rb*^*−/−*^ retina. Strikingly, we observed both IVP and DVP in the P18 *Rb/Bax* DKO retina (Fig. 5a–c). Quantification in the IVP revealed that *Bax*KO increased vessel coverage and length, and reduced lacunarity essentially to *WT* levels (Fig. 5d–f). The extent of the defects was greater in the *Rb*KO DVP; Bax loss did not restore vascularity to *WT* levels, but did elevate vessel coverage and length, and dramatically reduced lacunarity (Fig. 5d–f). In line with these cellular effects *Bax*KO rescued the reduction of *Fzd4*, *Norrin*, and *Tie2* expression in P7 *Rb*^*−/−*^ retina, and actually induced *Vegfa* and *Cxcr4* expression considerably by P18 (Fig. 2c, d). We also observed some increase in vasculature in the *Bax*^*−/−*^ relative to *WT* retina (Fig. 5c–e). These data suggest that the modest effects of *Bax* loss on natural neuronal pruning (Fig. 4b)^43^ promote vessel formation in the developing retina.

In summary, without reducing ectopic division, deleting *Bax* promotes survival of *Rb*^*−/−*^ cells, most prominently in the inner retina, which correlates with increased expression of angiogenic factors and dramatic rescue of the IVP and DVP.

### Inactivating Bax exposes new roles for Rb in retinal lamination

**Fig. 6:**
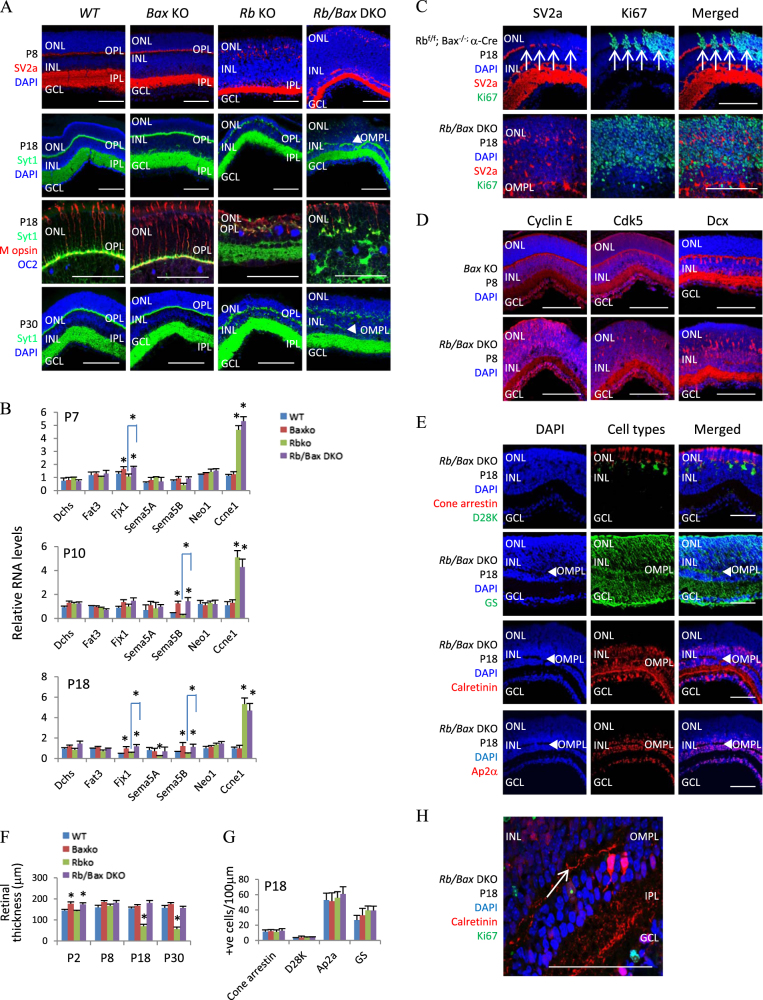
Lamination defects in the *Rb/Bax* DKO retina. **a** Retinal sections of indicated genotypes and ages were stained for nuclei (DAPI, blue), synaptic vesicles (SV2a, red; Syt1, green), cones (M opsin, red), and horizontal cells (OC2, blue). **b** RT-PCR analysis of the indicated genes and ages (*n* = 3). **c** Retinal sections of indicated genotypes and ages were stained for nuclei (DAPI, blue), division (Ki67, green), and synaptic vesicles (SV2a, red). Arrows indicate ectopically dividing cells disrupting the SV2^+^ OPL. **d** Retinal sections of indicated genotypes and ages were stained for nuclei (DAPI, blue), cyclin E (red), Cdk5 (red), and Dcx (red). **e** P18 *Rb/Bax* DKO retinal sections were stained for nuclei (DAPI, blue), cone (cone arrestin, red), horizontal (D28K, green), Müller (GS, green), or amacrine cells (Calretinin, red; Ap2a, red). Arrowheads highlight the OMPL separating the INL. **f** Retinal thickness for the indicated genotypes and ages. **g** Cell counts of cone (cone arrestin^+^), horizontal (D28K^+^), amacrine (Ap2a^+^), and Müller cells (GS^+^) of the indicated genotypes at P18. **h** P18 *Rb/Bax* DKO retinal sections were stained for nuclei (DAPI, blue), division (Ki67, green), and amacrine cells (Calretinin, red). Dendrites of Calretinin^+^ amacrine cell stratify in the IPL, as usual, but also the OMPL (arrow). Data are mean ± SD. Asterisks indicate significant difference between *WT* and other genotypes, or between *Rb*KO and *Rb/Bax* DKO as indicated by square brackets (*p < 0.05, one-way ANOVA followed by Bonferroni correction). ONL outer nuclear layer, OMPL outer misplaced plexiform layer, OPL outer plexiform layer, INL inner nuclear layer, IPL inner plexiform layer, GCL ganglion cell layer. Scale bar is 50 µm

The striking rescue of cell death in the *Rb/Bax* DKO retina also exposed two roles for Rb in retinal lamination. The first affected OPL formation. OPL forms in the P8 *WT* retina, but not in the *Rb*KO tissue until P18 (Figs. 1e and 4a, c, e). We assumed that this delay reflects the loss of rods and bipolar cells, and thus expected it to be rescued in the DKO retina (Fig. 4e–g). Unexpectedly, no OPL was evident in the DKO retina at P8 and P18 (Fig. 4a, c, e), which was confirmed by staining for synaptic vesicle proteins synaptotagmin 1 (Syt1)^44^ and synaptic vesicle glycoprotein 2a (SV2a)^45^ (Fig. 6a).

In contrast to the uniform OPL staining in P8 *WT* or *Bax*KO retina, SV2A^+^ synapses were disorganized in the *Rb*KO or DKO retinas (Fig. 6a). Syt1 staining was better ordered in the P18 *Rb*KO retina, even though the M opsin^+^ cones and Onecut2-positive (OC2^+^) horizontal cells were almost superimposed, but it remained disorganized in the DKO tissue, even though positioning of the cone and horizontal cells was restored (Fig. 6a). By P30, a Syt1^+^ OPL was evident in the DKO retina, although still less uniform than in other genotypes (Fig. 6a). In the *Rb*^*−/−*^ cortex, Neogenin 1 (Neo1) induction causes migration and lamination defects^25,40^. However *Neo1* was not induced in the *Rb*^*−/−*^ retina (Figs. 2b and 6b). Other migration regulators that were induced in the *Rb*^*−/−*^ cortex, including *Sema3d*, *ApoE*, *CCK*, *Twist1*, and *Twistnb*^25^, were also not altered in the *Rb*^*−/−*^ retina (Fig. 2b).

Potentially, ectopic division directly perturbs OPL formation, because its appearance correlated with a large reduction in ectopically dividing Ki67^+^ cells in the DKO retina between P18 and P30, and in the *Rb*KO retina between P8 and P18 (Fig. 4a). Furthermore, in the boundary between central α-Cre^−^ and peripheral α-Cre^+^ retina, OPL disruptions coincided precisely with Ki67^+^ cells (Fig. 6c, arrows), disordered SV2a vesicles coincide with dense Ki67 staining (Fig. 6c).

Cyclin E can bind and sequester cyclin-dependent kinase 5 (Cdk5), which plays important roles in neuronal migration and lamination by phosphorylating doublecortin (Dcx)^46–48^. As an E2f target, cyclin E increased in the *Rb*^*−/−*^ retina, while Cdk5 and Dcx levels had barely changed (Figs. 2b and 6b). In P8 *Bax*KO retina, Dcx and Cdk5 were most prominently expressed in the cell bodies and dendrites of horizontal and amacrine cells, while cyclin E was most evident in the OPL and less so the IPL and cytoplasm of some cells (Fig. 6d). In P8 *Rb/Bax* DKO retina the expression pattern of Dcx and Cdk5 was similar, but cyclin E was induced in many retinal cells (Fig. 6d). These data are consistent with the notion that cyclin E in ectopically dividing cells may interfere with Cdk5 function and contribute to the lamination defects in the *Rb*KO and *Rb/Bax* DKO retina.

In addition to delayed OPL formation, there was an unanticipated “outer misplaced plexiform layer” (OMPL) in P8 DKO retinas, which split the INL (Figs. 4a, c, e and 6a, e). It was not observed in either the *Rb*KO or *Bax*KO retinas (Fig. 6a). Excess cell production cannot explain this phenomenon since, at best, *Bax* loss restored the missing retinal cell types (Fig. 4f, g), retinal thickness was similar at all time points between DKO and control retinas, and *Bax* deletion had no effect on the numbers of *Rb*^*−/−*^ death-resistant cone, horizontal, amacrine, and Müller cells (Fig. 6e–g). Many amacrine cells flanked the OMPL, as indicated by Calretinin and Ap2α staining (Fig. 6e, h). A similar OMPL was observed in *Fat3*^*−/−*^, *Sema5A*^*−/−*^, and *Sema5B*^*−/−*^ retinas, in which amacrine cells are bipolar, leading to new synaptic contacts with bipolar cells^22,23^. Indeed, some amacrine cells in the DKO INL were bipolar and had ectopic dendrites that stratified in the OMPL (Fig. 6h, arrows).

In view of the phenotypic similarities between *Rb/Bax* DKO and *Fat3* or *Sem5* KO retinas, we assessed the mRNA levels of *Fat3* and *Sem5A & B*, as well as those of Dachsous (*Dchs*), a Fat3 ligand, and Four jointed box 1 (*Fjx1*), which modifies Fat activity. The levels of *Dchs*, *Fat3*, and *Sema5A* were not different between *WT*, *RbKO*, *BaxKO*, and DKO retina at three different time points, and while *Fjx1* and *Sema5B* were slightly increased in DKO retinas, this was also the case in the *Bax*KO control which has no OMPL (Fig. 6h). The occurrence of the OMPL in the DKO, where ectopic division is enhanced, and its rescue in the *Rb/E2f1* DKO retina, where ectopic division is blocked, suggest that it may also be linked to excess activity of cell cycle machinery. Irrespective, these data expose a new role for Rb in coordinating retinal lamination with phenotypic similarities to *Fat3*-deficient and *Sema5*-deficient retinas

## Discussion

Rb is critical in the retina as it promotes cell cycle exit, neuronal survival, SAC differentiation, and tumor suppression^26,27^. Here we exposed new roles in retinal angiogenesis and lamination. The vasculature was rescued by deleting *E2f1* or *Bax*, pinpointing neuronal loss rather than ectopic division in the phenotype. Rescuing death-prone neurons with *Bax* deletion exposed new roles for Rb in OPL and INL formation. These data expand our insight into the multi-faceted functions of Rb in retinal development.

To separate defects linked to division or death, we focussed on Bax because it mediates the death of *Rb/p107* DKO neurons in the brain^4^. Indeed, *Bax* loss rescued most rod bipolar and RGCs, and many rods, but had no major effect on ectopic cell division. Rb loss delays the formation of OPL from P8 to P18; we assumed this defect was due to rod and bipolar cell loss. However, while deleting *Bax* rescued many *Rb*^*−/−*^ rods and bipolar cells, it did not rescue the OPL defect. Despite the disorder, synaptic proteins were present, and Neogenin, which is induced and disrupts lamination in the *Rb/p10*7-null cortex^24,25^, was not elevated in the *Rb*-null retina.

The OPL defect may be related to ectopic cell division because *E2f1*KO promoted cell cycle exit and rescued OPL defects, but deleting *Bax*, which increased ectopic division due to extended cell survival, further delayed OPL genesis. Moreover, at the boundary of Cre expression, sporadic OPL disruption correlated perfectly with interspersed ectopically dividing cells. The eventual appearance of a narrow OPL correlated with delayed cell cycle exit at P18 or P30 in the *Rb*-null or *Rb/Bax*-null retina, respectively. Potentially, over-active cell cycle machinery disrupts formation of the synaptic layers. One candidate, cyclin E, can sequester Cdk5 which is important for lamination^48^, and we confirmed high levels of this E2f1 target in ectopically dividing *Rb*-null and *Rb/Bax*-null cells. Future work will address whether reducing cyclin E or elevating Cdk5 ameliorates the OPL defect.

The unnatural OMPL that split the *Rb/Bax*-null INL at P8 resembles the phenotype of *Fat3*^*−/−*22^, and *Sema5A*^*−/−*^*; Sema5B*^*−/−*23^ retinas, suggesting that *Rb* may affect these adhesive and repulsive cues^19^. We did not observe major changes in their expression. Cell cycle enzymes might also contribute to this phenotype as the OMPL separated post-mitotic amacrine cells in the inner INL from ectopically dividing amacrine cells in the outer INL (Fig. 4a).

Our work also exposes a critical role for Rb in the formation of intraretinal vasculature. In theory, Rb could influence angiogenesis through its positive effects on Hif1^14^, and/or by promoting survival of cells essential for vessel formation. Our results support the latter because rescuing death-prone *Rb*^*−/−*^ neurons with *Bax* deletion restored the IVP and DVP. Hif1 targets were relatively unaffected by Rb loss, perhaps due to induction of E2f7/8, known to augment Hif1 activity^15^, and instead other angiogenic regulators, such as *Norrin*, *Fzd4*, and *Tie2*, were down-regulated.

Excitatory retinal neurons, including RGCs and photoreceptors, can drive and regulate retinal angiogenesis^4,49^. Specifically, RGCs are required for the SVP development^9^, and photoreceptors are required for the DVP development^50^. Surprisingly, *Rb* deficiency did not affect the SVP development, even though *Rb*^*−/−*^ mouse retinas lose most RGCs^26^. In prior studies RGCs were depleted from the entire retina^9,10^, but in our case cell death is confined to the periphery, so conceivably the signals in the intact central retina are sufficient to drive the SVP development in the periphery.

Clearly, this was not the case for the IVP and DVP, which showed dramatic loss in the *Rb*-null retina. There is a very close relationship between the number of photoreceptors and vessel profiles in the DVP^50^. As there are still many rods in the *Rb*^*−/−*^ retina, their reduction may not explain all the vascular defects. Rod bipolar cells may affect the IVP development because these cells are essentially absent in *Rb*^*−/−*^ P18 retina^26^. *E2f1*KO rescued all the cell death of rods and bipolar cells, and completely rescued the vascular defects. *Bax* loss rescued about 80% of rod bipolar cells and 50% of rods, but rescued about 80% of IVP coverage and 50% of DVP coverage which, while not definitive, suggests a potential role for the bipolar cells.

Amacrine and horizontal cells are required for generating and maintaining the intraretinal vasculature^4^. However, in our study, amacrine and horizontal cells survive *Rb* deficiency, but intraretinal vascular plexi still could not develop, indicating that these inhibitory neuron-regulated pro-angiogenic factors are not sufficient for the retinal vascular development.

Our work exposes new roles for Rb in the developing retina, pinpointing aberrant E2f1 and Bax activity as key drivers of defects in neuronal survival and angiogenesis, and further implicating E2f1 in aberrant lamination. The Rb/E2f pathway is commonly involved in many diseases, such as most human cancers^13^, retinal degeneration^51^, diabetic retinopathy^52^, and neuronal degeneration^53^. Abnormal cell survival, proliferation, angiogenesis, and lamination are common features of these diseases. Thus, our findings will enhance our understanding of the pathogenesis and optimize the treatment strategies for these diseases in the future.

## Materials and methods

### Mouse strains and genotyping

Mice were treated according to institutional and national guidelines. All procedures were performed in compliance with the Association for Research in Vision and Ophthalmology statement for the use of animals in ophthalmic and visual research. *α-Cre* mice (P. Gruss), *Rb*^*f/f*^ mice (A. Berns), *E2f1*^*–/–*^ mice (M. Greenberg), *E2f2*^*–/–*^ mice (G. Leone), *E2f3*^*f/f*^ mice (G. Leone), *Z/Red* (Jackson Laboratory, stock#005438), and *Bax*^*−/−*^ (Jackson Laboratory, stock#002994) were maintained on a mixed (NMRI × C57/BL6 × FVB/N × 129sv) background.

*Bax-*null males are infertile, to generate *Rb*^*f/f*^;*Bax*^*−/−*^*;α-Cre* mice we first generated *Rb*^*f/f*^;*Bax*^*+/−*^*;α-Cre* males and *Rb*^*f/f*^;*Bax*^*−/−*^ females, and inter-bred them. Of 96 pups we obtained 50 with *α-Cre* and 48 with *Bax*^*−/−*^ alleles as predicted, but only 5 pups (5.2%) with *Bax*^*−/−*^*;α-Cre*, far less than the expected 24 pups (25%, Table 1), suggesting that *α-Cre* and *Bax*^*-*^ alleles may be on the same chromosome. We screened pups for a crossover event to generate *α-Cre* and *Bax*^*-*^ alleles on the same chromosome. We obtained *Rb*^*f/f*^;*Bax*^*−/−*^*;α-Cre* females, and bred them with *Rb*^*f/f*^;*Bax*^*+/−*^ males to generate experimental littermates.

**Table 1:** *Rb*^*f/f*^;*Bax*^*+/−*^*;α-Cre* males and *Rb*^*f/f*^;*Bax*^*−/−*^ females breeding offspring

Alleles	Predicted frequency	Observed frequency
*Rb* ^*f/f*^	100%	96/96 (100%)
*α-Cre*	50%	50/96 (52%)
*Bax* ^*−/−*^	50%	48/96 (50%)
*Bax* ^*−/−*^ *;α-Cre*	25%	5/96 (5.2%)

*Rb* retinoblastoma tumor suppressor, *f/f* floxed, *α-Cre* Pax6 alpha enhancer Cre

Mice of different genotypes were compared within the same litter and across a minimum of three litters. We have not noted any phenotypic differences in separate litters. Genotyping was performed as before^26,27,54^, and the primers for genotyping *Z/Red* mice are primers oIMR3847 and oIMR4110 for transgene (208 bp), and oIMR7338 and oIMR7339 for internal positive control (324 bp). The primers for genotyping *Bax*^*−/−*^ mice are primers oIMR0661 and oIMR0662 for mutant allele (507 bp), and oIMR0661 and oIMR0663 for WT allele (304 bp). The sequences of the above primers are listed in Jackson Laboratory genotyping protocols (www2.jax.org).

### Histology, immunofluorescence, and measurements

Eyeballs were fixed in 4% paraformaldehyde for 1 h at 4 °C, embedded in OCT (TissueTek 4583), frozen on dry ice, and cut into 12–14 µm sections on Superfrost slides. For immunohistochemistry, the retinal sections were dried at room temperature and incubated in blocking solution (0.5% normal donkey serum, 0.03% Triton X-100 in 1× phosphate-buffered saline (PBS)) for 1 h, then were incubated with primary antibodies such as active caspase-3 (Cell Signaling Technology, 9661), Ap2α (Santa Cruz, SC-8975), Brn3 (Santa Cruz, SC-6062), Calbindin (Sigma, C9484), Calretinin (Santa Cruz, SC-11644), Cdk5 (Santa Cruz, SC173), Cone arrestin (Millipore, AB15282), cyclin E (Upstate, 07-687), Dcx (Abcam, ab18723), glutamine synthetase (Millipore, MAB302), Ki67 (BD Science Pharmingen, 550609), M opsin (C.M. Craft and X. Zhu, University South California), Onecut2 (R&D System, AF6294), phospho-histone H3 (Santa Cruz, SC-8656), protein kinase Cα (Sigma, P5704), rhodopsin (Santa Cruz, SC-57433), SV2a (R. Janz, The University of Texas-Houston Medical School), and Syt1 (Abcam, ab13259). Vascular endothelial cells were labeled by fluorescein isothiocyanate (FITC)-conjugated isolectin B4 (Sigma, L2895). Antigen retrieval was performed as described by boiling sections in citric acid (H-3300, Vector Lab)^27^. Primary antibodies or labeled cells were visualized using donkey anti-mouse, donkey anti-rabbit, and donkey anti-goat antibodies conjugated with Alexa-488, Alexa-568, or Alexa-647 (1:1000; Molecular Probes). Nuclei were counter-stained with 4′, 6-diamidino-2-phenyindole (DAPI; Sigma) and mounted with Mowiol mounting medium.

For whole-mount staining, eyeballs were enucleated and incubated for 30 min in 4% paraformaldehyde in PBS. With a dissection microscope, a circumferential incision was made around the limbus, followed by removal of the anterior segment, lens, and vitreous body. The retinas were incubated at 4 ℃ with FITC-conjugated isolectin B4 (Sigma, L2895) and DAPI in PBS for 1–2 days. After briefly washing with PBS, radial cuts were made to divide the retina into four quadrants to flatten the retina, and flat retinas were mounted with Mowiol.

Stained sections and slides were analyzed using a Zeiss Axio Imager Z2 fluorescence microscope and Nikon C1si confocal microscope. Image J 1.50b with cell counter plugin (https://imagej.nih.gov/ij/) was used for cell counting following the online guide. The positive cells of active caspase-3, Ki67, and cell-type markers (including Brn3, PKCα) were counted manually. The thickness of ONLs was measured by the microscope program. For vascular blood vessel analysis, representative images were analyzed using the AngioTool software (https://ccrod.cancer.gov/confluence/display/ROB2/Home) to quantify the vessel coverage (percentage of area covered by IB4^+^ endothelial cells), average vessel length, and lacunarity (distribution of the gap sizes surrounding the object).

### Microarray dataset selection and analysis

The dataset GSE86372 at NCBI GEO database (http://www.ncbi.nlm.nih.gov/geo/) was used to compare *WT* vs. *RbKO* mouse P8 retinas, which include three *WT* and three *Rb*KO mouse P8 retinas. The data were analyzed by GEO2R from the GEO website. The genes, of which expression fold changes are >2 or <0.5, and adjusted *p* < 0.05 were selected as the *Rb*KO-related DEGs. Totally 677 DEGs were identified. The heatmap was generated using Heatmapper^55^. The function enrichment of DEG was performed using Enrichr^33,34^, the pathways with adjusted *p* < 0.05 were chosen to report.

### RNA extraction, RT, and quantitative real-time PCR

Total RNA was isolated from dissected peripheral retina using the TriPure isolation reagent (Roche, USA) or RNeasy mini kit (Qiagen) followed by digestion with RNase-Free DNase (DNA-free^TM^, Thermo Fisher Scientific) to remove DNA contamination. First-strand cDNA was synthesized from 0.2–0.5 µg of total RNA using the RT reagent kit with gDNA Eraser (TaKaRa, China) or SuperScript II first-strand synthesis system (Invitrogen). PCR primers are listed in Supplementary Table 1. Real-time quantitative PCR was performed using the qTOWER 2.2 PCR machine (Analytik Jena, Germany) or C1000 touch Thermal Cycler (Bio-Rad, USA). Tests were run in duplicate on three separate biological samples with EvaGreen PCR Supermix (SsoFastTM, Bio-Rad Laboratories, Singapore) or SYBRGreen PCR Master Mix (Applied Biosystems). PCR consisted of 40 cycles of denaturation at 95 °C for 15 s, and annealing and extension at 55 °C for 30 s. An additional cycle (95 °C, 15 s) generated a dissociation curve to confirm a single product. Values obtained for test RNAs were normalized to β-actin mRNA levels.

### Statistical analysis

All data were presented as mean ± SD. Statistical analysis was undertaken using the GraphPad Prism software (GraphPad Prism Software, Inc., San Diego, CA, USA). The results were analyzed by one-way analysis of variance (ANOVA) followed by Bonferroni correction for multiple comparisons. The threshold for significance was set at *p* < 0.05.

## Electronic supplementary material

Supplementary table 1
