# Supplementary material for: Rb is required for retinal angiogenesis and lamination
Source: Cell Death Dis. 2018 Mar 6;9(3):370. doi: 10.1038/s41419-018-0411-6 (PMC5840357; doi:10.1038/s41419-018-0411-6)
Supplement: Supplementary file 1 — Supplementary table 1 [file 41419_2018_411_MOESM1_ESM.docx]

**Supplementary Table 1: RT-PCR primer sequences**

| Genes | Forward primer (5’ to 3’) | Reverse primer (5’ to 3’) |
| --- | --- | --- |
| *Actb* | ACCACCACAGCTGAGAGGGA | GCCATCTCCTGCTCGAAGTC |
| *Bnip3* | AGGCGTCTGACAACTTCCAC | CCAAGGACCATGCTAGCTCT |
| *Ccne1* | CTCGGGTGTTGTAGGTTGCT | CTGTTGGCTGACAGTGGAGA |
| *Cxcr4* | TCTTCCTGCCCACCATCTAC | CCGTCATGCTCCTTAGCTTC |
| *Dchs1* | TGGCTACCTCTTCCCCCTAT | CCAGAGGTGGTTGTTCCACT |
| *E2f1* | CTGCAGCAACTGCAGGAGAG | CTCCGAAAGCAGTTGCAGC |
| *E2f7* | GTGCCTTGTGGCTGCTCCT | GCACAGAGTGAACGGACCG |
| *E2f8* | GCCTACCAGCTCACCCTACA | AGACTTCCAGTTTGCGCTGT |
| *Epo* | CTCCACTCCGAACACTCACA | CCTCTCCCGTGTACAGCTTC |
| *Fat3* | TAAACATGAACCAGGGCACA | ATGAGGTGCCTCTGGAATTG |
| *Fjx1* | CAGGCTGTTTCCTTTCCAAG | TCTTCGGATCCAATCTCCAC |
| *Fzd4* | GCCAATGTGCACAGAGAAGA | GGCAAACCCAAATTCTCTCA |
| *Id2* | AGGTGGAGCGTGAATACCAG | CAGCATTCAGTAGGCTCGTG |
| *Neo1* | GCCAAGGGAGTCTGTGATGT | CAATGCCTGTCAGTGGTGTC |
| *Norrin* | CACTGTGAGGAATGCAGCTC | TTTCTTGCCAGTCCGTCTCT |
| *Sema5a* | TCCTTGCAGTCACACTGGAG | GGCAGCAACTGAGGTAAAGC |
| *Sema5b* | TCACAGGACTCTTGGCACAG | GCCAGACTCTGCCTCCTATG |
| *Tie2* | GAGGCCGAACATTCCAAGTA | ATTGTCACATGGCCAAACAA |
| *Vegfa* | TCTTCCTGCCCACCATCTAC | TGGTAACCCATGACCAGGAT |
| *VegfR2* | AGTGGCTCTGTCCTCCAAGA | TCTCACCCATCCTCAACACA |
